# A Case of Takotsubo Cardiomyopathy After Mitral Valvuloplasty

**DOI:** 10.7759/cureus.43175

**Published:** 2023-08-08

**Authors:** Tomohiro Nakajima, Yutaka Iba, Tsuyoshi Shibata, Nobuyoshi Kawaharada

**Affiliations:** 1 Cardiovascular Surgery, Sapporo Medical University, Sapporo, JPN

**Keywords:** high age, dementia, iabp, mitral valvuloplasty, takotsubo cardiomyopathy

## Abstract

We present a case of an 82-year-old male patient with a history of severe mitral regurgitation, severe aortic regurgitation, chronic atrial fibrillation, and suicide attempts due to depression. The patient underwent mitral valvuloplasty and aortic valve replacement for mitral valve regurgitation and aortic valve regurgitation. The patient was extubated on the morning of the sixth postoperative day, but he was reintubated in the evening because of hypotension and an unstable respiratory status. Echocardiography revealed Takotsubo cardiomyopathy development, and the patient was treated with intra-aortic balloon pump (IABP) implantation, which was removed on postoperative day 11.

## Introduction

Takotsubo cardiomyopathy is a type of cardiomyopathy that presents with transient and characteristic cardiac dysfunction [1]. The pathogenesis of Takotsubo cardiomyopathy is still unknown, but sympathetic hyperfunction is the most likely explanation [2]. In most cases of Takotsubo cardiomyopathy, cardiac contractile abnormalities normalize within a few days to a few months. There are no clear data regarding prognosis; however, recovery generally occurs within one to two weeks. However, there are some severe cases in which affected individuals die of arrhythmia or heart failure while other cases experience recurrence [3].

## Case presentation

The patient was an 82-year-old male. He had symptoms of heart failure and underwent a thorough examination, which revealed severe mitral regurgitation due to mitral valve posterior leaflet P1 deviation, severe aortic regurgitation, and chronic atrial fibrillation. The patient had a history of cystectomy for bladder cancer, bladder fistula by ileal conduit, depression with a history of suicide attempts, and chronic renal failure (estimated glomerular filtration rate 30 mL/min). The patient requested to undergo surgery. Under general anesthesia and cardiac arrest, the patient underwent mitral valvuloplasty (P3 triangular resection), mitral annuloplasty with a 32-mm Physio Flex annuloplasty ring (Edwards Lifesciences, Irvine, CA,), aortic valve replacement with a 25-mm Inspiris (Edwards Lifesciences), and left coronary artery resection. The surgical duration was 449 minutes. We considered that the patient had delayed arousal because he had been taking antipsychotic medications.

**Figure 1:**
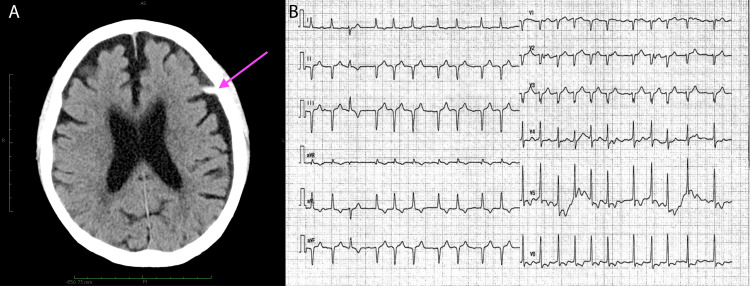
Head CT and ECG (A) Postoperative CT scan of the head showing a small amount of hemorrhage under the left subdural space. (B) On postoperative day 6, electrocardiography showed a decline in blood pressure after extubation. Negative inversion of the T wave was observed in leads I, aVl, and V5–6. CT, computed tomography

**Figure 2:**
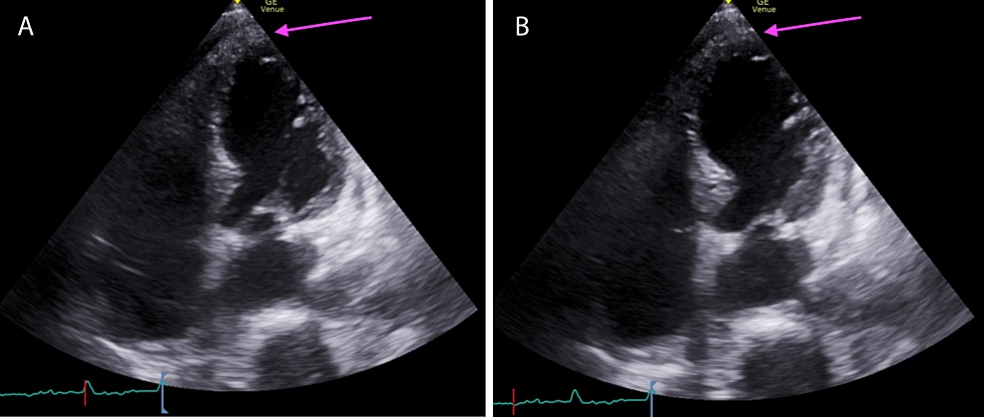
Echocardiogram Echocardiogram showed a decline in blood pressure after extubation on postoperative day 6. (A) Diastolic phase. (B) Systolic phase. The basal portion of the heart was contractile, but the apex was asystolic.

On the second postoperative day, computed tomography of the head revealed a mild subdural hemorrhage in the left temporal area (Figure 1A). The amount of hemorrhage was small and was not considered to be the cause of the patient’s prolonged loss of consciousness. There were no gross convulsions; therefore, we considered the patient to have an atypical absence seizure and administered anticonvulsants to awaken his consciousness. The patient was extubated on the morning of the sixth postoperative day, his heart rate was 130 beats per minute with atrial fibrillation, and his blood pressure gradually decreased. Electrocardiography showed a negative T wave, but there were no findings suggestive of ischemic heart disease (Figure 1B). Echocardiography showed apex asystole with an ejection fraction of 30% (Figures 2A, 2B). Coronary angiography revealed no evidence of organic stenosis or constriction in the right or left coronary arteries. A right heart catheter was placed and an intra-aortic balloon pump (IABP) was implanted. The patient was followed up until the signs and symptoms of Takotsubo cardiomyopathy had disappeared. The patient was managed in the intensive care unit without catecholamines. The cardiac index increased with each passing day and recovered to 2.0 on postoperative day 11. Tracheotomy was performed on postoperative day 14. The patient experienced postoperative delirium, which improved with time, and his respiratory condition gradually stabilized. On postoperative day 60, the patient was transferred to the hospital for continued rehabilitation. At five months postoperatively, the patient was able to speak with a speech cannula and was able to take small amounts of food by mouth.

## Discussion

In this case, a patient with depression and a history of suicide attempts underwent mitral valvuloplasty, aortic valve replacement, and left ventriculoplasty. The cause of Takotsubo cardiomyopathy has been reported to be diverse, but in this case, the patient developed Takotsubo cardiomyopathy due to postoperative cerebral hemorrhage, delirium, and the stress of intubation. The patient was in need of some form of assisted circulation. Considering that the acute phase of Takotsubo cardiomyopathy lasts approximately one week, we considered IABP, Impella, or extracorporeal membrane oxygenation (ECMO) to assist circulation during this period. IABP had the advantage of being the easiest to insert and not affecting the replaced aortic valve, but it had weak assisted circulation and may not achieve its purpose. Impella seemed to provide an adequate amount of supplemental circulation, but it had to pass through the replaced aortic bioprosthetic valve, which could cause mechanical damage to the aortic valve, which we wanted to avoid. He also had to use heparin, which carries the risk of contributing to cerebral hemorrhage [4]. ECMO appeared to provide adequate supplemental circulating volume, but had the disadvantage of increased afterload to the left ventricle, and required heparin, which could have contributed to cerebral hemorrhage. Considering these points, the final decision was made to implant an IABP, and if the patient continued to have a circulatory failure, to convert the patient to Impella. The patient was returned to the intensive care unit after IABP implantation. The patient's cardiac index was approximately 1.8, and he had no hemodynamic problems. On postoperative day 11, the IABP was removed. The patient had delirium, which made extubation difficult, and a postoperative tracheotomy was performed. The patient's cerebral hemorrhage improved without worsening and left no neurological sequelae. The patient was able to survive the acute phase with circulatory support by IABP and did not require heparin, thus eliminating the risk of contributing to cerebral hemorrhage. The patient had a multitude of problems at the same time, but each of them progressed without further deterioration.

## Conclusions

A patient with aortic valve replacement and postoperative cerebral hemorrhage developed Takotsubo cardiomyopathy. The patient required circulatory support. The need for the device to pass through the aortic valve and the use of heparin were considered. In the end, IABP was selected to overcome the acute phase of the disease.
